# Editorial: Complexity of tumor microenvironment: A major culprit in cancer development

**DOI:** 10.3389/fendo.2022.1059885

**Published:** 2022-10-20

**Authors:** Ihtisham Bukhari, Yuanwei Zhang, Rick Francis Thorne, Yang Mi

**Affiliations:** ^1^ Henan Key Laboratory of Helicobacter pylori, Microbiota and Gastrointestinal Cancers, Marshall Medical Research Center, Fifth Affiliated Hospital of Zhengzhou University, Zhengzhou, China; ^2^ Henan Provincial and Zhengzhou City Key Laboratory of Noncoding RNA and Cancer Metabolism, Henan International Join Laboratory of Non-coding RNA and Metabolism in Cancer, Translational Research Institute, Henan Provincial People’s Hospital, Academy of Medical Sciences, Zhengzhou University, Zhengzhou, Henan, China; ^3^ School of Life Sciences, University of Science and Technology of China, Hefei, Anhui, China; ^4^ School of Environmental and Life Sciences, University of Newcastle, Callaghan, NSW, Australia

**Keywords:** tumor microenvironment, cancer development, immune cell infiltration, prognosis, immune function

The tumor microenvironment (TME) is a complex landscape composed of intrinsic and extrinsic elements besides tumor cells including various immune cells, tumor-related stromal cells, and endothelial cells along with extracellular matrix components (1–3). Notably, the ability of tumor cells to invade surrounding tissues or metastasize through blood and lymphatic vessels implicitly involves cooperation with elements of the TME (4–6). In this regard, infiltrating immune cells such as T cells, B cells, macrophages, dendritic cells, monocytes, neutrophils, and mast cells have been associated with cancer development and progression (6–8). Additionally, these cells stimulate the host immune response by releasing cytokines, cytokine receptors, and other factors, which directly or indirectly promote or alternatively inhibit tumor cell proliferation (9, 10). Collectively these processes direct key events such as tumor recurrence, metastasis, and response to the immunotherapy (11, 12), thereby influencing clinical outcomes (13–15). However, the detailed profiles of immune cell infiltration and differentially expressed genes (metabolic, immune-related, or others) in many cancers continue to be elucidated (7, 16, 17). Several metabolic factors have shown an association with pathogenesis and progression of various cancers (18, 19) such as *de novo* lipid biosynthesis is a crucial regulator in the prostate cancer (20–22). An anecdotal observation metabolic health of the individuals may influence the prognosis and treatment of the cancers.

Indeed, support for this intriguing hypothesis is gaining momentum, for example, androgen deprivation therapy (ADT) reduces testosterone in the body which inhibits the prostate cancer (23–25). However, studying constituents of the TME can help understand the underlying mechanisms of cancer development and progression (26–29). It has been well established that metabolic factors and infiltrating cells in TME potentially serve as prognostic markers in various cancers (30, 31). Recent advances and improvements in cancer therapy have shifted the treatment focus toward hormonal therapy and immunotherapy such as immune checkpoint inhibitors (ICIs) and chimeric antigen receptor (CAR) T-cell adoptive immunotherapy. The latter involves manipulating T cells in the laboratory to add artificial receptors that can invoke attacks against cancer cells (32–34). However, CAR-T therapy remains limited by the lack of appropriate targets in solid tumors (33, 35–37). However, advanced-stage patients or those presenting with unfavorable tumors inevitably face disease progression with dire outcomes (38–40). Thus, more comprehensive studies related to the genetic regulation of tumors through metabolic and endocrine factors, immune cell infiltration, and immune functions are urgently required to identify the underlying mechanisms of cancer development and progression towards improved biomarkers and/or applications of targeted therapy.

The current Research Topic aimed to collect studies reporting advancements in clinical and basic research related to the tumor microenvironment and regulation of cancers through metabolic, endocrine factors, and immune cells. After a rigorous review process, the current volume presents an authoritative collection of twelve articles exploring new dimensions in this research field.

First, the review by Aguilar-Cazares et al. provides a systematic account of the current literature describing the roles of inflammatory mediators within the tumor microenvironment, particularly their dynamics in growing tumors. Here inflammatory factors including IL-6, IL-1, TNF-α, G-CSF, and GM-CSF produced by cancer cells and stromal cells make essential contributions to cancer-associated inflammation. Ye et al. further describe the activation of inflammation in diabetic pancreatic cancer patients *via* the infiltration of CD8+T cells into the TME, which intriguingly acts to reduce tumor growth and metastasis. Similarly, Huang et al. using single-cell analysis of pancreatic cancer developed a four-gene predictive model which also indicated the differential infiltration of memory B-cell subtypes into the TME.

Treating solid tumors is always challenging with surgical resection, chemotherapy and radiotherapy being the longstanding options. However, the resurgence of immunotherapy over recent years, for example, involving CAR-T cells has shown promising results in hematological malignancies. Gastrointestinal cancers such as hepatocellular carcinoma (HCC) are some of the most lethal cancers and generally, patients with such solid tumors have not presently benefited from CAR-T approaches. The review by Guizhen et al. investigates the factors preventing CAR-T success in HCC, dissecting the evidence for why the TME represents a significant barrier to the infiltration, survival and activity of CAR-T cells. Taking cues from other cancer types, they conclude that modifying CAR-T cells may help their persistence in the TME or otherwise combinatorial approaches such as combining CAR-T with immune checkpoint inhibitors. Moreover, Dong et al. showed that cancer-associated fibroblast (CAF) related genes are significantly associated with immune regulation in HCC. Patients with tumors showing higher CAF gene expression were resistant to chemotherapy (cisplatin and doxorubicin) and tyrosine kinase inhibitors (TKI) (sorafenib) with worse overall survival.

Similarly, Zhang et al. used the TKI Aumolertinib to treat a lung cancer case with brain metastasis coupled with Osimertinib-induced cardiotoxicity. The patient showed significant recovery after Aumolertinib treatment with negligible adverse effects recorded, suggesting clinical outcomes could be improved by supplementing TKIs with CAR-T or other immunotherapies. A further enlightening report linking the TME with cancer prognosis by Li et al. presented evidence for the differential expression of immune-related genes in lung cancer. They found that Ribonucleotide Reductase Regulatory Subunit M2 (RRM2) represented a potential new metabolic checkpoint and therapeutic target. Ge et al. also reported the prognostic significance of pyroptosis-derived lncRNAs from lung adenocarcinoma tissues, which could be used as alternative therapeutic targets.

Yan et al. constructed an eight-gene model for low and high-risk prognostication in diffuse large B cell lymphoma (DLBCL) patients, interestingly showing that low-risk cases exhibited a proinflammatory immune cell-enrichment profile. An alternative DNA damage repair gene signature predicting survival in cutaneous melanoma patients revealed by Liang et al. showed that positive benefits were associated with immune cell enrichment in the TME together with the expression of immune checkpoint-related genes. Additionally, Jiang et al. considered PET/CT a promising method for early diagnosing lesions in patients with biochemical recurrence of prostate cancer. Finally, Wang et al. showed how hypoxic conditions enhance the stemness and proliferative capacity of rat peripheral blood-derived mesenchymal stromal cells (PBMSCs), a finding of interest to tissue engineering but also in cancer models.

## Conclusion and prospects

The tumor microenvironment is a unique tissue landscape that must be understood if therapeutic strategies against cancer are to be successful. Twelve contributions to this topic highlight different aspects of how TME affects cancer outcomes. Most studies report the identification of molecular markers that may be potentially used as diagnostic, therapeutic, and prognostic targets. Altogether, these studies increase our biological understanding of cancer and tumor microenvironment aspects but especially highlight the cruciality of PDL1, CAR-T, and TKIs in cancer treatment. It can be anticipated that these contributions will find broad applications, ranging from purely scientific endeavors to clinical guidelines for cancer treatment.

## Author contributions

All authors listed have made a substantial, direct, and intellectual contribution to the work and approved it for publication.

## Conflict of interest

The authors declare that the research was conducted in the absence of any commercial or financial relationships that could be construed as a potential conflict of interest.

## Publisher’s note

All claims expressed in this article are solely those of the authors and do not necessarily represent those of their affiliated organizations, or those of the publisher, the editors and the reviewers. Any product that may be evaluated in this article, or claim that may be made by its manufacturer, is not guaranteed or endorsed by the publisher.
